# A Randomized Trial Assessing the Muscle Strength and Range of Motion in Elderly Patients following Distal Radius Fractures Treated with 4- and 6-Week Cast Immobilization

**DOI:** 10.3390/jcm10245774

**Published:** 2021-12-09

**Authors:** Jarosław Olech, Grzegorz Konieczny, Łukasz Tomczyk, Piotr Morasiewicz

**Affiliations:** 1 Provincial Specialist Hospital in Legnica, Orthopedic Surgery Department, Iwaszkiewicza 5, 59-220 Legnica, Poland; o.jaroslaw@yahoo.com; 2 Faculty of Health Sciences and Physical Education, Witelon State University of Applied Sciences, Sejmowa 5A Street, 59-220 Legnica, Poland; gkonieczny@wp.pl; 3 Department of Food Safety and Quality Management, Poznan University of Life Sciences, 60-624 Poznan, Poland; lukasz.tomczyk@up.poznan.pl; 4 Department of Orthopaedic and Trauma Surgery, University Hospital in Opole, Institute of Medical Sciences, University of Opole, al. Witosa 26, 45-401 Opole, Poland

**Keywords:** distal radius, fracture, muscle strength, grip strength, range of motion, aging

## Abstract

Background: There is no consensus among orthopedic surgeons as to the required period of cast immobilization in distal radius fractures in elderly patients. The purpose of this study was to assess muscle strength and range of motion symmetry in elderly patients after distal radius fractures with different periods of cast immobilization. Methods: This study evaluated 50 patients (33 women and 17 men), aged over 65 years, after cast immobilization treatment for distal radius fracture. The mean age at the beginning of treatment was 71 years. The mean duration of follow-up was 1 year and 3 months. The first subgroup (*n* = 24) comprised the patients whose fractures had been immobilized in a cast for 6 weeks, another subgroup (*n* = 26) comprised the patients with 4-week cast immobilization. We assessed: (1) muscle strength, (2) range of motion. Results: The mean grip strength in the treated limb was 71% and 81% of that in the healthy limb in the groups with 4-week and 6-week cast immobilization, respectively (*p* = 0.0432). The study groups showed no differences in the mean grip strength in the treated limbs or the mean grip strength in the healthy limbs. The mean treated limb flexion was 62° and 75° in the 4-week and 6-week immobilization groups, respectively (*p* = 0.025). The evaluated groups showed no differences in terms of any other range of motion parameters. The grip strength and range of motion values were significantly lower in the treated limb than in the healthy limb in both evaluated groups. Only the values of wrist radial deviation in the 6-week cast immobilization group showed no differences between the treated and healthy limbs. Conclusion: Higher values of injured limb muscle strength and greater mean range of wrist flexion were achieved in the 6-week subgroup. Neither of the evaluated groups achieved a symmetry of muscle strength or range of motion after treatment. Full limb function did not return in any of the elderly distal radius fracture patients irrespective of cast immobilization duration.

## 1. Introduction

Distal radius fractures (DRFs) pose a serious problem due to their high incidence [1,2,3,4,5,6,7,8,9,10,11,12,13,14,15]. The risk of DRF has been reported as 9–139/10,000 people per year [3,4,8,11,12,15]. These fractures most commonly involve the distal radial epiphysis, which is estimated to be the site of 15–21% of all fractures. This is also the third most common location of osteoporotic fractures [1,2,3,4,5,6,7,8,9,10,11,12,14].

In elderly patients with poor bone quality and poor condition of the adjacent soft tissues, with only slight radial deformity and shortening and a fracture morphology that justifies conservative treatment, the preferred treatment method is closed reduction with cast immobilization [11,13,15,16,17]. In elderly patients Kilic prefers treatment via immobilization in a cast [16]. Moreover, elderly patients have shown good clinical and functional outcomes with cast immobilization [11].

One aspect of DFR treatment regarding which there is no consensus is the duration of cast immobilization [9,13,15,16,17,18], with some orthopedic surgeons advocating for 4 weeks [9,16], some for 5 weeks [13,16,18], and some for up to 6 weeks [9,17] of immobilization. The issue of muscle strength and range of motion in elderly patients with DRF treated via cast immobilization for different lengths of time has not been fully evaluated.

Long-term cast immobilization after DRF adversely affects muscle strength, range of motion, and limb function [14,15,18,19]. Thus, on the one hand, shortening the period of cast immobilization after DRF should be beneficial, on the other hand, a shorter period of cast immobilization may lead to nonunion and bone fragment displacement.

Arora et al. compared range of motion and muscle strength in patients aged over 65 years with DRF treated with a cast and those treated with a volar locking plate [13]. Those authors reported a lack of difference between the groups in terms of range of motion, whereas muscle strength was greater in the volar locking plate group [13]. Egol reported greater muscle strength in elderly patients with DRF treated surgically, in comparison with that in patients treated with a cast, and a greater degree of supination in patients treated with cast immobilization [20]. Zengin observed greater muscle strength in elderly patients with DRF treated with plate fixation than that in cast-immobilized patients, with no difference between the groups in terms of range of motion [17]. None of those authors evaluated muscle strength and range of motion in elderly patients following cast immobilization of varied duration.

We hypothesized that the duration of limb immobilization in a cast affects the symmetry of both muscle strength and range of motion in elderly patients following DRF treatment.

Due to the lack of a broader analysis of this important issue, the purpose of our study was to assess the symmetry of functional parameters following DRF treatment with two different cast immobilization periods.

## 2. Materials and Methods

This was a prospective study. Over the period from June 2020 to November 2020, there were 117 patients treated at our center for DRFs. The study inclusion criteria were: a DRF treated with closed reduction and cast immobilization; age of 65 years or older; follow-up of at least 1 year after treatment completion; available complete medical records regarding the treatment; and complete data on range of motion and grip strength assessment. The exclusion criteria were: a compound fracture; treatment with other methods, such as external fixation, plate fixation, or K-wire fixation; age under 65 years; incomplete treatment records (i.e., patients who continued their treatment elsewhere); and incomplete data on range of motion or grip strength. All patients had been informed that study participation was completely voluntary. The study had been approved by the local review board.

The study inclusion criteria were met by 50 patients (33 women and 17 men). The mean age at the beginning of treatment was 71 years (ranging from 65 to 86 years). The mean duration of follow-up was 1 year and 3 months (ranging from 1 year to 1 year and 6 months).

Once the diagnoses had been established and written informed consent obtained, the patients were randomized into two groups (cast immobilization for 4 or 6 weeks) with the use of sequentially numbered, closed envelopes.

The patients, who were stratified by the period of DRF cast immobilization, formed two study subgroups. One subgroup (*n* = 24) were the patients treated with cast immobilization for 6 weeks, and the other subgroup (*n* = 26) were the patients who underwent closed reduction and cast immobilization for 4 weeks.

All patients included in the study underwent emergency room closed reduction and immobilization in a below-elbow cast. In the entire study group there were no cases of a secondary bone fragment displacement that would require surgical correction.

All patients underwent outpatient radiographic follow-up after 5–7 days, 4–6 weeks, and in 3-month intervals thereafter. Outpatient clinical and radiographic follow-up visits were scheduled in 2–6-week intervals.

The plaster casts were removed after 4 or 6 weeks, depending on the study group. For the first 3–6 weeks following cast removal, the patients were advised to use the limb sparingly and were assigned finger and wrist exercises. Limb loading was increased gradually, based on the progress of bone remodeling at the fracture site, as assessed radiographically, as well as based on clinical symptoms. Wrist and finger strengthening exercises were introduced at 3–6 weeks.

The following functional parameters were assessed: (1) muscle strength, (2) range of motion.

Muscle strength (grip strength), expressed in kilograms, was assessed with a Smedley Hand Dynamometer (GIMA). The grip strength in the uninjured (healthy) hand was compared with that in the treated limb, with the result expressed as the percentage of the grip strength measured in the healthy limb [7,9,17].

Range of motion was measured with a goniometer and included: wrist flexion, extension, abduction (radial deviation), and adduction (ulnar deviation), with the results expressed in degrees [7,9,17].

All these parameters were evaluated at a follow-up visit at least 1 year after treatment completion. One experienced orthopedist performed dynamometer and goniometer measurements.

The study subgroups (representing different cast immobilization periods) were compared in terms of the individual functional parameters.

The data were analyzed statistically using Statistica 13.1. The Shapiro–Wilk test was used to check for normality of distribution. Student’s *t*-test was used to compare variables assuming a normal distribution. The Mann–Whitney test was used in the case of variables assuming a different order than the normal one. The level of statistical significance was set at *p* < 0.05.

## 3. Results

The individual subgroups did not differ in terms of mean patient age (*p* = 0.5628) (Table 1).

**Figure 1:**
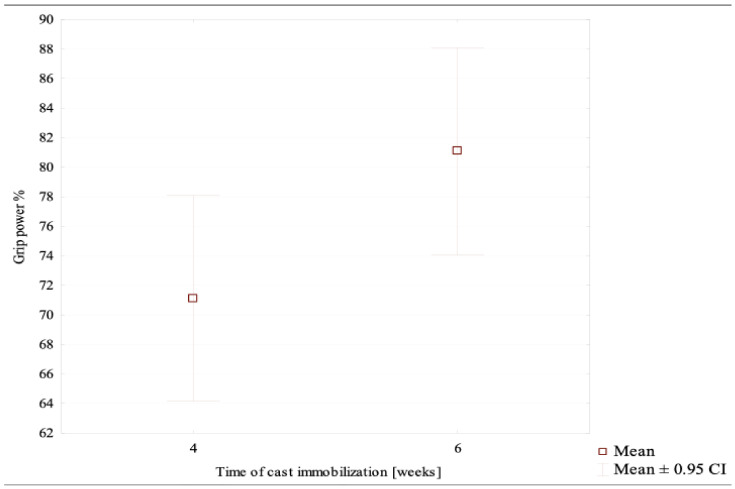
The mean relative grip strength values in the 4-week and 6-week subgroups.

Our study showed the mean grip strength following DRFs in all patients to be approximately 76% of that in the healthy limb. The mean relative grip strength values in the 4-week and 6-week subgroups were 71% and 81%, respectively. These differences were statistically significant (*p* = 0.0432) (Table 1, Figure 1).

**Figure 2:**
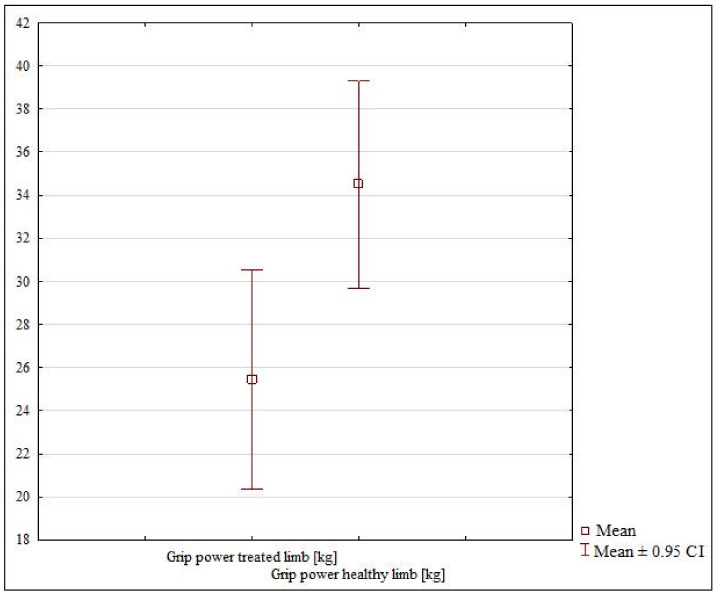
The grip strength in the healthy and treated limb in the 4-week subgroup.

**Figure 3:**
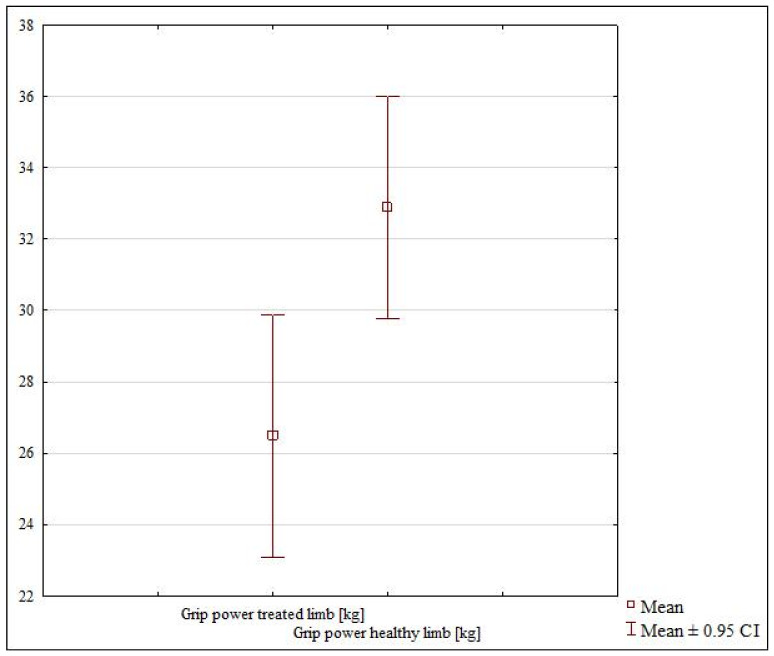
The grip strength in the healthy and treated limb in 6-week subgroup.

The 4-week cast immobilization subgroup showed a lack of grip strength symmetry between the healthy and treated limb (*p* = 0.01047) (Table 2, Figure 2). The patients from the 6-week subgroup also showed a lack of grip strength symmetry between the uninjured and injured limb (*p* = 0.06218) (Table 2, Figure 3).

**Figure 4:**
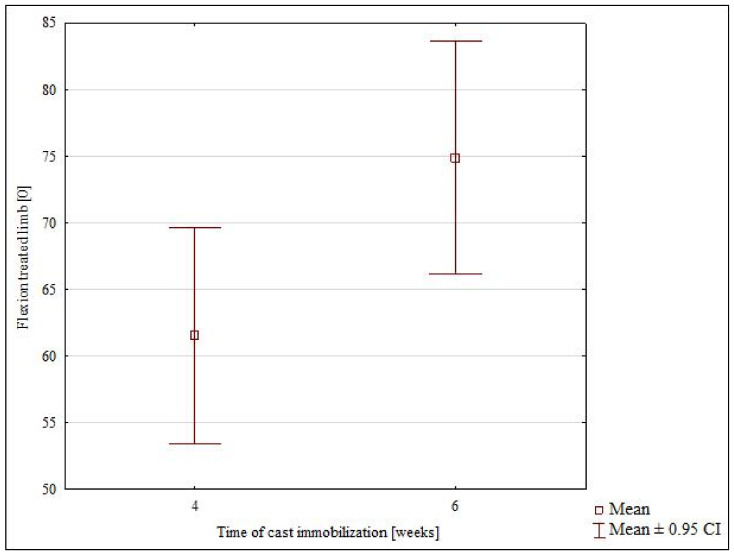
The range of flexion in the 4-weeks and 6-week subgroups.

The mean wrist flexion measured in all study subjects was 67.9° in the treated limb and 84.4° in the healthy limb. The best range of flexion (74.9°) was achieved in the 6-week subgroup, and the most limited flexion (61.5°) was observed in the 4-week subgroup. These differences were significant (*p* = 0.025) (Table 1, Figure 4). The 4-week immobilization subgroup exhibited significant differences in wrist flexion between the healthy and treated limb (*p* = 0.00001) (Table 2). The 6-week immobilization subgroup also showed significant differences in wrist flexion between the healthy and treated limb (*p* = 0.00002) (Table 2).

The mean wrist extension in the 4-week subgroup was 50.1°, whereas in the 6-week subgroup it was 57°; the difference was not statistically significant (Table 1). Wrist extension asymmetry was observed in the 4-week subgroup (*p* = 0.00073) (Table 2). The range of wrist extension in the healthy and treated limb was also asymmetrical in the 6-week subgroup (*p* = 0.004961) (Table 2).

The mean radial deviation was 18.6° and 21.2° in the patients whose fractures were immobilized in a cast for 4 weeks and 6 weeks, respectively; this difference was not statistically significant (Table 1). The 4-week subgroup showed significant differences in terms of mean radial deviation between the uninjured and injured limb (*p* = 0.0258) (Table 2). Conversely, there were no significant differences in terms of mean radial deviation in the healthy and treated limbs in patients treated with a cast for 6 weeks (*p* = 0.06597) (Table 2).

The mean ulnar deviation in the 4-week subgroup was 33.3°, whereas in the 6-week subgroup it was 39.5°; this difference was not statistically significant (Table 1). However, there was a significant difference in terms of mean ulnar deviation between the healthy and treated limb of patients who had worn a cast for 4 weeks, which demonstrates an asymmetry of this parameter (*p* = 0.00224) (Table 2). The 6-week subgroup showed differences in the mean ulnar deviation between the healthy and treated limb (*p* = 0.000547) (Table 2). There were no differences in complications in the two assessed groups. There was no need for changing casts in any of the evaluated patients.

## 4. Discussion

This study aimed to verify whether a shorter period of cast immobilization (4 weeks) in elderly DRF patients would help them complete the treatment and resume their normal activities earlier than those treated with 6 weeks of cast immobilization. So far, there have been no studies assessing the functional outcomes of cast immobilization treatment of varied duration in elderly DRF patients. We observed a significantly greater grip strength in the treated limb (81% of the grip strength in the healthy limb, on average) and a greater range of flexion in the treated limb (75° on average) in our 6-week subgroup than in the 4-week subgroup. Our study groups showed no differences in terms of any other evaluated parameters. Thus, the results of our study support our hypothesis only to some extent. 

The distal radial epiphysis is one of the most common fracture locations [1,2,3,4,5,6,7,8,9,10,11,12,13,14,15], and the treatment of these fractures is an important part of patient management in any trauma/orthopedic ward. Stable extra-articular fractures, particularly those in elderly patients, can be treated via closed reduction with immobilization in a plaster cast [11,13,15,16,17,19]. 

Assessing multiple parameters following orthopedic treatment is important from the point of view of orthopedic and rehabilitation specialists and the patients themselves [7,8,9,13,14,15,17,20]. Apart from the radiographic and biomechanical assessment, functional assessment seems to be of great significance, as it is the post-treatment limb function that is of crucial importance to patients, doctors, and rehabilitation specialists alike. Some authors have demonstrated good functional outcomes, despite worse radiographic outcomes, in DRF patients treated with cast immobilization, in comparison with those treated surgically [9,11,17]. Conversely, some authors reported a relationship between good radiographic and good clinical outcomes following DRF treatment [5,21,22]. 

There is no consensus among orthopedic surgeons as to the required period of cast immobilization in DRF [9,13,15,16,17,18]. Various authors prefer an immobilization period of 4 weeks [9,16], 5 weeks [13,16,18], or 6 weeks [9,17]. A long period of cast immobilization may reduce the post-treatment range of motion and muscle strength in the limb [14,15,18,19]. A shorter period of cast immobilization, provided bone union has been achieved, may allow for earlier rehabilitation as well as earlier use and resumed function of the limb [14,15,17,18]. Theoretically, muscle strength and range of motion should be comparable (symmetrical) in both upper limbs [15,17,19]. Achieving statistically similar (i.e., symmetrical) values of muscle strength and range of motion in the treated and healthy limbs following DRF treatment indicates good clinical and functional outcomes [15,19]. 

Katayama et al. reported a mean grip strength of 84.6% of that in the healthy limb in patients with osteoarthritis following DRF treatment with volar plate fixation [7]. Lameijer et al. reported a mean grip strength of 79% of that measured in the uninjured limb [8]. Toon et al. observed a mean grip strength of 83.29% of that in the unaffected limb in patients who underwent DRF internal fixation with a volar plate and 81.26% in patients treated with a plaster cast [9]. Kilic reported a mean grip strength of 57.3% of that in the healthy limb in DRF patients managed with plaster cast immobilization [16]. In a study by Zengin et al. the patients treated with a volar locking plate and those treated with a plaster cast exhibited a mean grip strength of 67.7% and 57.5% of that in the uninjured limb, respectively [17]. Arora et al. assessed muscle strength in patients over 65 years of age with DRF treated with cast immobilization or volar locking plate fixation [13]. Twelve months after the fracture, those authors observed a greater muscle strength in the volar locking plate subgroup (22.2 kg) in comparison with that in the plaster cast subgroup (18.8 kg) [13]. Egol reported a greater muscle strength in elderly DRF patients treated surgically (17.7 kg) than in those treated with cast immobilization (12.7 kg), whereas the range of supination was greater in the plaster cast subgroup [20]. Other authors reported a mean grip strength of 19 kg in 67 treated DRF patients [14]. In our study, there were no differences in grip strength between the study groups. Our study results are similar to those reported in the literature [7,8,9,13,14,16,17]. We observed a lack of symmetry between the healthy and treated limb in terms of grip strength in both groups of patients. This indicates incomplete return of pre-fracture limb function after DRF in elderly patients irrespective of the duration of cast immobilization. The pain or discomfort that persisted following treatment completion may have additionally reduced post-treatment muscle strength and intensified grip strength asymmetry.

The mean range of wrist extension reported by Lameijer et al. was 53° [8]. Toon et al. observed a mean wrist extension of 67.5° in their group of DRF patients managed with volar plate fixation and 72.9° in their plaster cast group [9]. Arora et al. compared range of motion in 73 patients over 65 years of age with DRF treated with cast immobilization or volar locking plate fixation [13]. Those authors reported a lack of differences in terms of range of motion between these two groups [13]. Zengin observed no differences in range of motion between the elderly patients with DRF treated with plate fixation and those treated with a plaster cast [17]. The range of wrist extension in our patients was similar in both evaluated subgroups; these results are consistent with those reported in the literature [8,9,13]. In our study, we observed wrist extension asymmetry between the healthy and treated limb in both evaluated groups. In terms of wrist flexion, the mean range reported by Lameijer et al., was 52° [8]. Toon et al. reported mean wrist flexion of 63.1° in their DRF patients managed with a volar plate and 64.1° in the group managed with a plaster cast [9]. Arora et al. reported a mean wrist flexion of 57° in elderly patients following 5 weeks of cast immobilization [13]. In our study, the greatest significant difference between treated subgroups was in the range of wrist flexion with the 6-week subgroup (74.9°) compared with the 4-week subgroup (61.5°); these results are consistent with those reported in the literature [8,9,13]. We noted a wrist flexion asymmetry between the healthy and treated limb in both groups of patients. Lameijer et al. observed a mean radial deviation of 14° [8]. Toon et al. reported a mean radial deviation of 15.6° in their DRF group managed with a volar plate and 15.7° in patients immobilized with a plaster cast [9]. Our results were consistent with those reported in the literature [8,9,13]. In our study, the range of radial deviation in the healthy limb was comparable with that in the treated limb in patients treated with cast immobilization for 6 weeks. A study by Lameijer et al. demonstrated a mean ulnar deviation of 23° [8]. Toon et al., reported a mean ulnar deviation of 22.8° in DRF patients treated with a volar plate and 17.9° in the cast immobilization group [9]. In our study there were no differences in the range of ulnar deviation between the two evaluated subgroups. We observed an ulnar deviation range asymmetry between the healthy and treated limb in both evaluated subgroups. Range of motion limitations and range of motion asymmetry between the healthy and treated limb suggest incomplete return of limb function in elderly DRF patients. Longer rehabilitation protocols should be considered particularly in patients treated with 4-week cast immobilization. The observed asymmetry in the evaluated parameter values may have been associated with a certain level of pain persisting after treatment completion.

The shorter cast immobilization period in the elderly may have produced incomplete bone remodeling at the fracture site. This, in turn, may have increased pain and worsened limb function in comparison with those parameters in the 6-week subgroup.

The greatest muscle strength and greatest mean range of wrist flexion in the 6-week immobilization subgroup may be due to the fact that the patients from this subgroup resumed their daily activities earlier, while the patients from the other (4 week-) subgroup were inhibited by fears of destabilizing their fracture, and to the fact that the proportion of patients who resumed exposing the fractured limb to normal load bearing was the highest in the 6-week immobilization subgroup. Well-chosen, intensive, individualized, and long-lasting rehabilitation may improve upper limb function following DRF treatment in elderly patients.

Our study has several limitations. Firstly, the sample size was relatively small. Nevertheless, other authors have also conducted their studies in similar-sized or even smaller groups of patients [7,9,14,16,17,18]. Secondly, the study did not have control participants who did not have a DRF. In the future, we plan to study with control participants who did not have a DRF. The strengths of our study include a uniform rehabilitation protocol, patient randomization, the individual groups showing no differences in terms of patient age, examinations being conducted by one surgeon following a single protocol.

## 5. Conclusions

We observed incomplete return of full limb function after DRF in elderly patients, irrespective of the duration of cast immobilization.

We observed an asymmetry in terms of grip strength and range of motion between the affected and unaffected limb in both evaluated subgroups.

Greater muscle strength in the affected limb (though not quite equal to that in the uninjured limb) and greater mean range of wrist flexion was achieved in the 6-week subgroup.

Our study indicates that elderly patients with DRF managed with cast immobilization should undergo longer and more intensive rehabilitation regimens.

## Figures and Tables

**Table 1:** Detailed results of the grip power and range of motion of individual subgroups.

Analyzed Variable (Mean ± Standard Deviation)	4-Week Group *n* = 26	6-Week Group *n* = 24	*p* Value
age of patients (years)	71.34 ± 4.99	72.20 ± 5.46	0.5628 *
grip power (%)	71.12 ± 14.24	81.07 ± 12.59	0.0321 **
grip power treated limb (kg)	25.45 ± 12.53	27.6 ± 11.46	0.5317 *
grip power healthy limb (kg)	34.48 ± 11.92	31.17 ± 9.69	0.2893 *
flexion treated limb (degrees)	61.53 ± 9.1	74.87 ± 10.66	0.025 *
flexion healthy limb (degrees)	84.46 ± 13.1	84.37 ± 13.7	0.9818 *
extension treated limb (degrees)	50.17 ± 17.47	57.02 ± 17.34	0.1711 *
extension healthy limb (degrees)	66.38 ± 14.92	58.6 ± 12.47	0.0521 *
ulnar deviation treated limb (degrees)	33.25 ± 13.22	39.54 ± 15.41	0.127 *
ulnar deviation healthy limb (degrees)	45.38 ± 13.93	46.7 ± 11.47	0.7167 *
radial deviation treated limb (degrees)	18.59 ± 11.7	21.18 ± 15.31	0.5026 *
radial deviation healthy limb (degrees)	27.76 ± 16.67	22.56 ± 14.3	0.2436 *

* student’s *t*-test. ** Mann–Whitney U test.

**Table 2:** The grip power and range of motion symmetry in the 4-week group and 6-week group.

**Analyzed Variable (Mean ± Standard Deviation)**	**Treated Limb**	**Healthy Limb**	***p* Value**
4-Week Group			
grip power (kg)	25.45 ± 8.53	34.48 ± 9.32	0.01124 **
flexion (degrees)	61.53 ± 14.1	84.46 ± 13.1	0.00001 *
extension (degrees)	50.17 ± 12.47	66.38 ± 14.92	0.00073 *
ulnar deviation (degrees)	33.25 ± 13.21	45.38 ± 12.63	0.00224 *
radial deviation (degrees)	18.59 ± 9.12	27.76 ± 10.32	0.0258 *
**Analyzed Variable (Mean ± Standard Deviation)**	**Treated Limb**	**Healthy Limb**	***p* Value**
6-Week Group			
grip power (kg)	26.48 ± 9.96	32.89 ± 8.92	0.005213 **
flexion (degrees)	67.94 ± 15.21	84.42 ± 12.32	0.00002 *
extension (degrees)	53.46 ± 12.58	62.65 ± 10.21	0.004961 *
ulnar deviation (degrees)	36.27 ± 11.52	46.02 ± 10.69	0.000547 *
radial deviation (degrees)	19.84 ± 13.47	25.27 ± 15.64	0.06597 *

* student’s *t*-test. ** Mann–Whitney U test.

## Data Availability

The data presented in this study are available on request from the corresponding author. The data are not publicly available due to privacy.
